# Psychological distress among dialysis patients during the COVID-19 Omicron pandemic: risk and protective factors across hemodialysis and peritoneal dialysis

**DOI:** 10.3389/fpsyt.2026.1710260

**Published:** 2026-05-18

**Authors:** Ching-Yi Chang, Shang-Feng Tsai, Li-Ling Huang, Mu Chi Chung, Shih-Yun Chen, Yi-Hua Chou, Huan-Yi Lin, Cheng-Hsu Chen, Te-Feng Yeh

**Affiliations:** 1 Department of Nursing, Taichung Veterans General Hospital, Taichung, Taiwan; 2 Division of Nephrology, Department of Internal Medicine, Taichung Veterans General Hospital, Taichung, Taiwan; 3 Department of Life Science, Tunghai University, Taichung, Taiwan; 4 Department of Post−Baccalaureate Medicine, College of Medicine, National Chung Hsing University, Taichung, Taiwan; 5 Department of Healthcare Administration, Central Taiwan University of Science and Technology, Taichung, Taiwan; 6 Division of Clinical Toxicology, Department of Medical Toxicology, Taichung Veterans General Hospital, Taichung, Taiwan

**Keywords:** COVID-19, dialysis, hemodialysis, insomnia, peritoneal dialysis, protective factors, psychological distress, risk factors

## Abstract

**Introduction:**

This study investigated the prevalence and severity of psychological distress among Taiwanese dialysis patients during the COVID-19 Omicron outbreak, compared differences between hemodialysis (HD) and peritoneal dialysis (PD) patients, and identified risk and protective factors associated with mental health.

**Methods:**

A cross-sectional survey was administered to 245 dialysis patients (117 HD, 128 PD) at an academic medical center in central Taiwan. Data collected included demographic and clinical characteristics, the Depression, Anxiety, and Stress Scale-21 (DASS-21), the Insomnia Severity Index (ISI), and the Pandemic Worsening Index (PWI). Statistical analyses involved descriptive statistics, generalized linear modeling (GLM), and stepwise regression to identify significant predictors.

**Results:**

Insomnia was the most prevalent symptom (54.7%), followed by stress (41.2%), anxiety (40.4%), and depression (14.3%). PD patients reported significantly greater psychological distress than HD patients. Regression analyses identified unemployment, reduced household income, comorbidities, and unhealthy behaviors (smoking and alcohol use) as significant risk factors. Conversely, complete vaccination and independence in daily activities were significant protective factors.

**Conclusions:**

Both HD and PD patients experienced substantial psychological burdens during the pandemic, though their stressors and coping mechanisms differed. Routine psychological assessment should be integrated into dialysis care, particularly for patients facing socioeconomic hardship or multiple comorbidities. Timely supportive interventions, together with strategies to maintain functional independence, ensure full vaccination coverage, and strengthen social safety nets, may help address psychological distress and enhance resilience in dialysis populations during public health crises.

## 1 Introduction

Since the onset of the COVID-19 pandemic in late 2019, global public health systems and individuals with chronic illnesses have faced unprecedented challenges. The emergence of the Omicron variant in late 2021, characterized by high transmissibility and immune evasion, marked a new phase of the pandemic. Although Omicron infections were generally milder and associated with lower hospitalization and mortality rates than earlier variants, the overwhelming number of cases placed severe strain on healthcare systems worldwide, particularly affecting unvaccinated individuals and high-risk populations with chronic comorbidities or immunocompromised conditions (1, 2).

Taiwan initially controlled community transmission through strict border measures and public health interventions, resulting in only 823 confirmed cases and 9 deaths in 2020. However, in April 2021, the Alpha variant led to a surge of 14,574 cases and 829 deaths within two months, despite the implementation of nationwide Level 3 alert measures (3). In contrast, the Omicron outbreak in 2022 proved far more severe. From April to July, over 4.56 million cases and 12,707 deaths were reported, representing the highest cumulative toll since the beginning of the pandemic (4, 5).

Patients with chronic kidney disease (CKD), particularly those receiving hemodialysis (HD) or peritoneal dialysis (PD) for end-stage renal disease (ESRD), were disproportionately vulnerable during the pandemic. Their increased risk was attributed to impaired immunity, multimorbidity, and the intensive, ongoing nature of dialysis treatment. Uremic conditions and long-term dialysis further suppress immune function, while comorbidities such as hypertension, diabetes, and cardiovascular disease independently elevate the risk of severe COVID-19 outcomes and mortality (6–9).

Beyond infection risk, dialysis patients also endured significant psychological burdens. Frequent hospital visits and prolonged exposure to healthcare personnel heightened infection-related anxiety, while disruptions in healthcare delivery, transportation challenges, and social isolation exacerbated uncertainty and distress. Some patients reported delaying medical care or concealing symptoms to avoid quarantine, reinforcing feelings of fear and helplessness (10, 11). For PD patients, home-based isolation and reduced provider contact weakened social support networks, leading to loneliness and, in severe cases, deterioration of family relationships (11, 12).

Psychological distress is well recognized as highly prevalent among individuals with CKD, especially those receiving dialysis. Prior research has consistently documented elevated rates of depression, anxiety, and emotional distress in CKD populations, even before the emergence of COVID-19 (13). During the pandemic, this pre-existing vulnerability appeared to intensify, as multinational studies reported increased psychological symptoms among dialysis patients (10). More recent evidence further emphasizes the complex interplay between biological frailty, treatment dependence, and psychosocial stressors in shaping mental health outcomes in CKD, underscoring the multifactorial nature of psychological burden in this population (14).

Cumulative stressors rendered dialysis patients not only medically but also psychologically vulnerable. Perceptions of pandemic severity have been shown to amplify mental health problems, with higher perceived risk strongly associated with greater depression, anxiety, and stress (14, 15). During the 2022 Omicron surge, the unprecedented spike in infections—together with residual trauma from Taiwan’s 2021 Level 3 alert—likely intensified fears of infection, hospitalization, and death, thereby exacerbating psychological distress.

Accordingly, this study aimed to evaluate the psychological distress of Taiwanese dialysis patients during the peak of the Omicron outbreak, with specific attention to depression, anxiety, stress, and insomnia. The Pandemic Worsening Index (PWI) was applied to assess perceived pandemic severity and its association with psychological outcomes. Comparisons were made between HD and PD patients, and associations with demographic, clinical, and vaccination-related variables were explored. Findings from this study provide essential evidence to guide the development of tailored psychosocial interventions to alleviate psychological burdens in this highly vulnerable population.

## 2 Material and methods

### 2.1 Study setting and approval

This study was conducted in accordance with the Taiwan Human Subjects Research Act and approved by the Institutional Review Board of Taichung Veterans General Hospital (IRB No. CE21527A). All participants provided verbal informed consent after being informed of their rights, including the option to withdraw at any time. To ensure confidentiality, completed questionnaires and consent forms were sealed by the researcher, and all data were collected anonymously.

**Figure 1:**
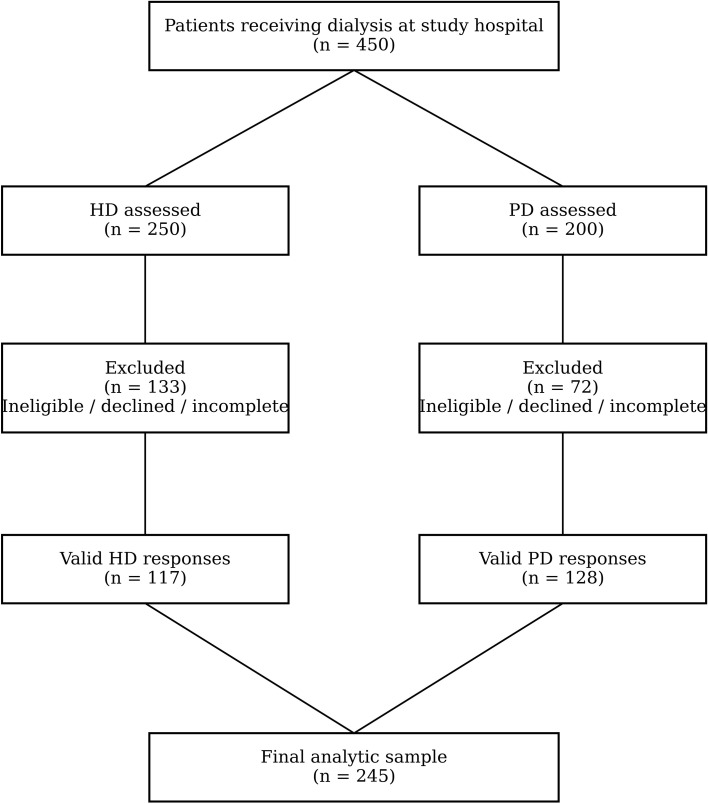
Flow diagram of patient selection and enrollment.

The study was conducted at an academic medical center in central Taiwan that manages approximately 250 hemodialysis (HD) and 200 peritoneal dialysis (PD) patients. The number of patients fluctuates slightly over time as new patients are admitted and existing patients transfer to other facilities. HD treatments are performed in the hospital two to three times per week, while PD patients visit the center once per month for routine evaluation, laboratory testing, and medication or supply exchange. The HD program is supported by 13 attending nephrologists who conduct monthly ward rounds, 4 chief residents who supervise each dialysis session, and 46 dialysis nurses responsible for in-center hemodialysis care. The PD program is managed by 10 attending nephrologists who provide regular outpatient consultations and 7 specialized PD nurses who deliver patient education and home-based follow-up. Eligibility criteria were: (1) adult patients aged 20 years or older, (2) regular receipt of dialysis at the study hospital, and (3) ability to independently complete the questionnaire. Patients who were unable to communicate effectively due to mental or linguistic limitations were excluded. After excluding 45 ineligible HD patients, 200 HD and 200 PD questionnaires were distributed. Ultimately, 117 valid responses were obtained from HD patients and 128 from PD patients, corresponding to response rates of 58.5% and 64.0%, respectively. The patient selection and enrollment process is illustrated in **Figure 1**.

### 2.2 Measures

A structured questionnaire was administered, incorporating the Depression, Anxiety, and Stress Scale-21 (DASS-21), the Insomnia Severity Index (ISI), and items assessing patients’ sociodemographic and clinical characteristics.

#### 2.2.1 Depression, anxiety and stress scales 21

The DASS-21 is a validated instrument for assessing symptoms of depression, anxiety, and stress. It comprises 21 items, with seven items allocated to each subscale. Responses are rated on a 4-point Likert scale (0–3), and subscale scores are summed and multiplied by two to yield final scores. Each subscale is categorized into five severity levels: normal, mild, moderate, severe, and extremely severe (17, 18).

To capture patients’ perceptions of pandemic severity, this study incorporated a supplementary question asking whether the Omicron outbreak was perceived as more severe than Taiwan’s 2021 Level 3 alert. Each affirmative response was assigned a score of 1, and the Pandemic Worsening Index (PWI) was calculated as the sum of these responses. A weighted total score, reflecting the extent to which psychological symptoms were affected by the pandemic, was derived by multiplying each subscale score by the corresponding PWI. Prior research has confirmed the reliability of the DASS-21 in evaluating psychological status among dialysis patients (19).

#### 2.2.2 The insomnia severity index

The Insomnia Severity Index (ISI) is a validated instrument for assessing the severity and impact of insomnia. It comprises seven items rated on a 5-point Likert scale (0 = no problem to 4 = very severe problem), yielding a total score ranging from 0 to 28. Severity is classified as follows: 0–7 = no clinically significant insomnia, 8–14 = subthreshold or mild insomnia, 15–21 = moderate insomnia, and 22–28 = severe insomnia (20, 21).

To assess the perceived impact of the pandemic, this study added a supplementary item to each ISI question, asking whether symptoms worsened during the Omicron outbreak compared with Taiwan’s 2021 Level 3 alert. Each affirmative response was assigned a score of 1, and the Pandemic Worsening Index (PWI) was calculated as the sum of these responses. A weighted total score was further derived by multiplying the ISI score by the PWI, thereby reflecting the extent to which insomnia symptoms were perceived as exacerbated by the pandemic.

#### 2.2.3 Personal characteristics of the patients

The personal characteristics of dialysis patients were categorized into three groups: sociodemographic characteristics, dialysis and clinical conditions.

Sociodemographic characteristics included gender, age, education level, marital status, number of children, participation in religious activities, employment status, number of family members living together, availability of a separate room, cohabitation with healthcare or long-term care workers, daily activity status, primary source of income, whether they were the main earner in the household, and whether their household income was impacted during the COVID-19 pandemic.

Dialysis and clinical conditions included the modality of dialysis (HD or PD), dialysis vintage, history of regular visits for significant non-renal diseases, smoking habits, alcohol consumption, mode of transportation, primary companions, and number of COVID-19 vaccine doses received. Alcohol consumption was assessed using a five-level question on drinking habits; however, because 86% of participants reported never drinking, responses were dichotomized into non-drinkers and drinkers for analysis.

The PD cohort included both automated peritoneal dialysis (APD) and continuous ambulatory peritoneal dialysis (CAPD) modalities: 41 patients (32.0%) used CAPD only, 25 (19.5%) used APD only, and 62 (48.4%) used both. Because the study focused on comparing overall psychological distress between HD and PD, these subgroups were combined for analysis. Subgroup analyses showed no significant differences in DASS-21 or ISI outcomes between APD and CAPD, except for a lower raw depression score in APD patients.

### 2.3 Statistical analysis

Before scale score computation, missing item responses were handled using mean imputation at the individual scale level to minimize incomplete domain scores and preserve the integrity of construct-level analyses. Personal characteristics and the severity categories of the DASS-21 and ISI were summarized as frequencies and percentages. Continuous variables, including DASS-21 scores, ISI scores, the Pandemic Worsening Index (PWI), and weighted scores, were expressed as means with standard deviations. Differences in categorical characteristics between HD and PD patients were examined using chi-square tests. Generalized linear models (GLM) with a normal distribution and identity link function were applied to compare DASS-21 and ISI scores between HD and PD patients after adjusting for personal characteristics that showed significant differences between the two groups. Stepwise multiple regression analysis was conducted to identify factors independently associated with DASS-21 and ISI outcomes among dialysis patients. All personal characteristics, including sociodemographic variables, dialysis-related factors, and clinical conditions, were initially entered into the models. Variables that did not achieve statistical significance were sequentially removed, and only those meeting the predefined significance criterion were retained in the final models. The distributional assumptions of the outcome variables were examined using skewness and kurtosis statistics. All original scores, PWI scores, and ISI-related weighted scores generally met the assumption of normality, whereas only the DASS weighted scores showed mild deviation from normality. For the final stepwise regression models of the DASS weighted scores, diagnostic tests were further performed. Standardized residual histograms and normal P–P plots indicated that residuals were approximately normally distributed, and scatterplots of standardized residuals against standardized predicted values showed no evident funnel-shaped pattern or systematic trend, supporting the assumption of homoscedasticity. Therefore, the final regression models were considered to meet the basic assumptions of linear regression. All analyses were performed using SPSS Statistics version 26.0 (IBM Corp., Armonk, NY, USA), and a two-tailed p value < 0.05 was considered statistically significant.

## 3 Results

### 3.1 Comparing personal characteristics among dialysis patients

**Table 1** summarizes the demographic and clinical characteristics of the 245 dialysis patients enrolled in this study, including 117 HD and 128 PD patients. Overall, 54.7% were male, the largest age group was 50–64 years (40.0%), and nearly two-thirds (64.5%) were married. The distribution of the number of patients with children was balanced, with 31.4% reporting none, 36.7% reporting one or two, and 31.8% reporting three or more. Regarding employment, 40.8% were employed, 41.2% unemployed, and 18.0% unable to work. Most patients (74.7%) reported independence in daily activities, 87.8% had a separate room at home, and 71.0% were economically self-sufficient. More than half (50.2%) lived with at least three family members. With respect to dialysis vintage, the largest proportion (34.7%) had received dialysis for 2–5 years, and 49.0% required regular hospital visits for other major illnesses. For transportation, 51.0% relied on self-transportation, and 58.0% had received four doses of the COVID-19 vaccine.

**Table 1:** Comparing personal characteristics among dialysis patients.

Personal characteristics	Overall	Dialysis modality		χ2	p
		HD	PD		
Total	245	117	128		
Gender				3.243	0.072
Female	111(45.3)	46(39.3)	65(50.8)		
Male	134(54.7)	71(60.7)	63(49.2)		
Age				28.953	0.000
Under 49 years old	73(29.8)	18(15.4)	55(43)		
50–64 years old	98(40)	48(41)	50(39.1)		
65 years old and above	74(30.2)	51(43.6)	23(18)		
Marital status				4.481	0.106
Married	158(64.5)	82(70.1)	76(59.4)		
Unmarried	61(24.9)	22(18.8)	39(30.5)		
Divorced, Separated, Widowed	26(10.6)	13(11.1)	13(10.2)		
Number of children				23.614	0.000
None	77(31.4)	24(20.5)	53(41.4)		
Less than 2	90(36.7)	39(33.3)	51(39.8)		
3 or more	78(31.8)	54(46.2)	24(18.8)		
Participation in religious activities				6.960	0.031
Not at all	57(23.3)	19(16.2)	38(29.7)		
Rarely	94(38.4)	46(39.3)	48(37.5)		
Some participation	94(38.4)	52(44.4)	42(32.8)		
Employment status				13.849	0.001
Employed	100(40.8)	34(29.1)	66(51.6)		
Unemployed	101(41.2)	55(47)	46(35.9)		
Unable to work	44(18)	28(23.9)	16(12.5)		
Availability of a separate room in the household	215(87.8)	103(88)	112(87.5)	0.016	0.899
Living with healthcare or long-term care workers	29(11.8)	15(12.8)	14(10.9)	0.208	0.649
Number of family members living together				0.567	0.753
No one	15(6.1)	8(6.8)	7(5.5)		
Less than 2	107(43.7)	53(45.3)	54(42.2)		
3 or more	123(50.2)	56(47.9)	67(52.3)		
Daily activities independently	183(74.7)	73(62.4)	110(85.9)	17.926	0.000
Economically self-sufficient	174(71)	81(69.2)	93(72.7)	0.348	0.555
Main source of household income	85(34.7)	35(29.9)	50(39.1)	2.258	0.133
Impact of the pandemic on household income				7.070	0.070
Not at all	84(34.3)	41(35)	43(33.6)		
Slight	97(39.6)	44(37.6)	53(41.4)		
Moderate	40(16.3)	25(21.4)	15(11.7)		
Severe	24(9.8)	7(6)	17(13.3)		
Tenure of dialysis				13.768	0.003
Less than 2 years	52(21.2)	16(13.7)	36(28.1)		
2–5 years	85(34.7)	36(30.8)	49(38.3)		
6–10 years	51(20.8)	30(25.6)	21(16.4)		
More than 11 years	57(23.3)	35(29.9)	22(17.2)		
Regular hospital visits for other major illnesses	120(49)	68(58.1)	52(40.6)	7.487	0.006
Smoking habits	15(6.1)	4(3.4)	11(8.6)	2.848	0.091
Alcohol consumption	35(14.3)	17(14.5)	18(14.1)	0.011	0.917
Mode of transportation for dialysis				12.627	0.002
Self-transportation	125(51)	47(40.2)	78(60.9)		
Family pick-up and drop-off	53(21.6)	27(23.1)	26(20.3)		
Taking public transportation	67(27.3)	43(36.8)	24(18.8)		
Companion during dialysis				4.699	0.095
no one	116(47.3)	53(45.3)	63(49.2)		
familiy	116(47.3)	54(46.2)	62(48.4)		
others	13(5.3)	10(8.5)	3(2.3)		
Number of doses of vaccination received				18.978	0.000
Within 2 doses	39(15.9)	19(16.2)	20(15.6)		
3 doses	64(26.1)	16(13.7)	48(37.5)		
4 doses	142(58)	82(70.1)	60(46.9)		

Comparative analysis revealed significant differences between HD and PD patients (p < 0.05). HD patients were older, with 43.6% aged ≥65 years, whereas 43.0% of PD patients were <49 years. A higher proportion of HD patients had three or more children (46.2%), while 41.4% of PD patients had none. Unemployment was more prevalent among HD patients (47.0%), whereas PD patients were more frequently employed (51.6%). Independence in daily activities was reported more often by PD than HD patients (85.9% vs. 62.4%). Dialysis vintage also differed: HD patients were more likely to have been on dialysis for 6–10 years (25.6%) or ≥11 years (29.9%), while PD patients more often had <2 years (28.1%) or 2–5 years (38.3). Regular hospital visits for non-renal illnesses were more common among HD patients (58.1% vs. 40.6%). In terms of transportation, HD patients more frequently relied on public transportation (36.8%), whereas PD patients were more likely to use self-transportation (60.9%). Finally, vaccination coverage differed significantly, with a higher proportion of HD patients having received four doses compared with PD patients (70.1% vs. 46.9%).

### 3.2 Comparison of DASS-21 and ISI scores between HD and PD patients

**Figure 2:**
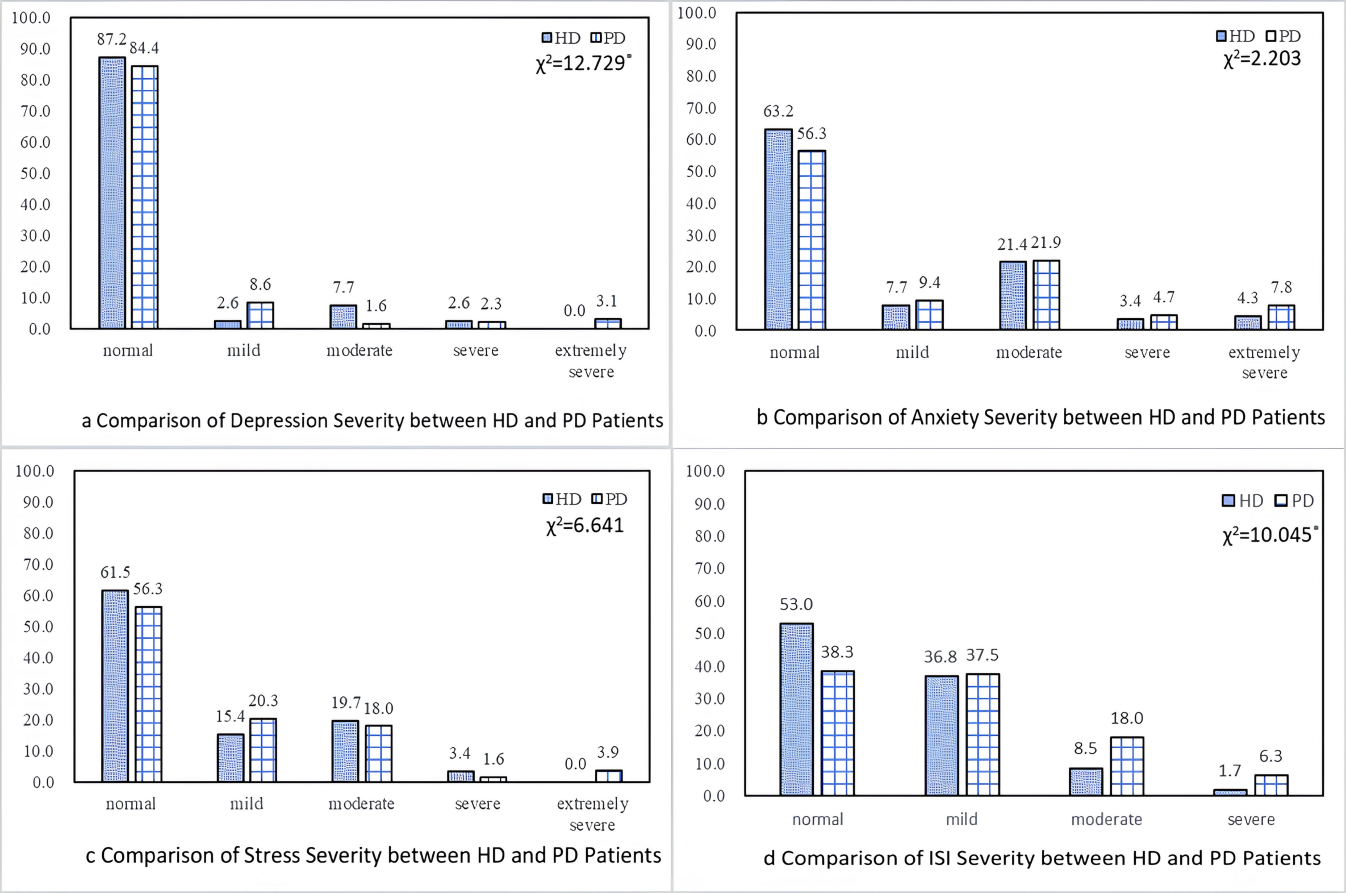
**(a)** Comparison of depression severity between HD and PD patient. **(b)** Comparison od anxiety severity between HD and PD patients. **(c)** Comparison of stress severity between HD and PD patients. **(d)** Comparison of ISI severity between HD and PD patients.

As shown in **Table 2** and **Figure 2**, the mean scores for depression, anxiety, stress, and insomnia among all dialysis patients were 8.01 (SD = 7.83), 7.02 (SD = 6.53), 8.07 (SD = 7.40), and 9.23 (SD = 5.72), respectively. The prevalence of symptoms ranging from mild to extremely severe was 14.3% for depression, 40.4% for anxiety, 41.2% for stress, and 54.7% for insomnia, with insomnia emerging as the most common complaint. The score distributions are further illustrated in **Supplementary Figure 1**.

**Table 2:** Comparison of DASS-21 and ISI scores between HD and PD patients.

Variables	Overall	Unadjusted		t	Adjusted		F
		HD	PD		HD	PD	
	Mean(SD)	Mean(SD)	Mean(SD)		Mean(SD)	Mean(SD)	
DASS-21							
Depression							
Score	8.01(7.83)	7.81(7.16)	8.19(8.42)	-0.374	9.30(0.86)	10.74(0.82)	1.730
PWI	1.09(1.97)	0.77(1.74)	1.38(2.12)	-2.449^*^	1.09(0.22)	1.80(0.21)	6.315^*^
Weighted Score	18.43(45.50)	12.91(34.55)	23.48(53.23)	-1.860	20.16(5.11)	33.89(4.84)	4.486^*^
Anxiety							
Score	7.02(6.53)	6.27(5.80)	7.70(7.08)	-1.719	7.23(0.73)	9.50(0.69)	6.084^*^
PWI	1.02(1.72)	0.55(1.37)	1.45(1.88)	-4.284^***^	0.8(0.19)	1.77(0.18)	16.004^***^
Weighted Score	13.88(32.53)	7.59(21.78)	19.63(39.11)	-3.009^**^	11.57(3.69)	25.41(3.50)	8.741^**^
Stress							
Score	8.07(7.40)	7.3(6.78)	8.77(7.87)	-1.555	8.58(0.84)	10.30(0.80)	2.597
PWI	1.2(1.96)	0.85(1.82)	1.52(2.02)	-2.757^**^	1.07(0.22)	1.87(0.21)	7.962^**^
Weighted Score	18.43(41.40)	12.56(32.14)	23.80(47.85)	-2.173^*^	18.28(4.71)	31.82(4.47)	5.125^*^
ISI							
Score	9.23(5.72)	8.12(4.97)	10.24(6.18)	-2.975^**^	9.20(0.64)	11.58(0.61)	8.508^**^
PWI	1.21(2.14)	0.91(1.71)	1.48(2.45)	-2.097^*^	1.06(0.25)	1.76(0.24)	4.888^*^
Weighted Score	19.5(40.82)	12.93(28.59)	25.51(48.77)	-2.487^*^	16.70(4.73)	31.53(4.49)	6.099^*^

PWI, pandemic worsening index Weigjted Score=Score*PWI.

^*^ p<0.05; ^**^: p<0.01; ^***^: p<0.001.

The adjusted variables included dialysis modality, age, number of children, participation in religious activities, employment status, daily free activities, companion during dialysis, mode of transportation for dialysis, and companion during dialysis.

Regarding the Pandemic Worsening Index (PWI), mean values were 1.09 (SD = 1.97) for depression, 1.02 (SD = 1.72) for anxiety, 1.20 (SD = 1.96) for stress, and 1.21 (SD = 2.14) for insomnia. The corresponding weighted scores were 18.43 (SD = 45.50), 13.88 (SD = 32.53), 18.43 (SD = 41.40), and 19.50 (SD = 40.82). The score distributions are further illustrated in **Supplementary Figure 2**.

In unadjusted analyses, PD patients demonstrated significantly higher PWI and weighted scores than HD patients across all domains: depression (PWI: 1.38 vs. 0.77, p < 0.05), anxiety (1.45 vs. 0.55, p < 0.001), stress (1.52 vs. 0.85, p < 0.01), and insomnia (1.48 vs. 0.91, p < 0.05). Weighted scores followed the same pattern. After adjusting for personal characteristics, PD patients continued to exhibit significantly higher PWI and weighted scores for depression, anxiety, stress, and insomnia (all p < 0.05). These findings indicate that PD patients bore a consistently greater psychological burden than HD patients, independent of demographic and clinical differences.

### 3.3 Related factors of DASS-21 and ISI scores among dialysis patients

As shown in **Table 3**, stepwise regression analysis identified multiple personal and clinical characteristics significantly associated with depression, anxiety, stress, and insomnia among dialysis patients.

**Table 3:** Stepwise regression analysis of the factors related to DASS-21 and ISI between HD and PD patients.

Variables	Score		PWI		Weighted score	
	b(95%CI)		b(96%CI)		b(97%CI)	
Depression						
constant	9.855(7.324,12.387)	^***^	1.737(1.196,2.279)	^***^	10.958(0.625,21.291)	^*^
Some participation in religious activities (Not at all)					13.716(0.913,26.520)	^*^
Unable to work (Employed)	2.743(0.251,5.234)	^*^				
Daily activities independently (No)	-2.908(-5.119,-0.696)	^*^	-0.578(-1.117,-0.039)	^*^		
Severe impact of the pandemic on household income (Slight)	4.998(1.906,8.09)	^**^	1.309(0.512,2.106)	^**^	36.516(18.473,54.56)	^***^
Regular hospital visits for other major illnesses (No)	1.989(0.115,3.864)	^*^			15.259(4.642,25.877)	^**^
Smoking habits (No)			1.224(0.238,2.21)	^*^	24.368(1.612,47.124)	^*^
4 doses of vaccination received (3 doses)	-2.816(-4.664,-0.967)	^**^	-0.73(-1.205,-0.255)	^**^	-14.25(-25.036,-3.465)	^*^
R^2^	0.169		0.127		0.162	
Adj R^2^	0.151		0.113		0.145	
F	9.694	^***^	8.752	^***^	9.267	^***^
Anxiety						
constant	10.730(8.765,12.695)	^***^	1.885(1.372,2.398)	^***^	1.94(-10.618,14.498)	
HD(PD)			-0.973(-1.395,-0.552)	^***^	-9.495(-17.353,-1.636)	^*^
Unable to work (Employed)	-1.602(-3.171,-0.033)	^*^				
Availability of a separate room in the household					13.673(2.191,25.156)	^*^
Daily activities independently (No)	-2.972(-4.748,-1.196)	^**^	-0.704(-1.185,-0.223)	^**^		
Severe impact of the pandemic on household income (Slight)	5.298(2.719,7.878)	^***^	1.243(0.560,1.926)	^***^	30.936(18.284,43.588)	^***^
Regular hospital visits for other major illnesses (No)					8.934(1.318,16.55)	^*^
Alcohol consumption (No)					14.162(3.334,24.99)	^*^
4 doses of vaccination received (3 doses)	-2.327(-3.874,-0.780)	^**^			-8.552(-16.353,-0.752)	^*^
R2	0.155		0.151		0.190	
Adj R2	0.141		0.140		0.170	
F	11.033	^***^	14.294	^***^	9.308	^***^
Stress						
constant	8.524(7.047,10.001)	^***^	1.332(0.948,1.717)	^***^	34.014(21.298,46.73)	^***^
HD(PD)					-12.385(-22.767,-2.003)	^*^
Unable to work (Employed)	2.916(0.590,5.242)	^*^				
Daily activities independently (No)					-18.385(-30.195,-6.576)	^**^
Severe impact of the pandemic on household income (Slight)	3.883(0.878,6.888)	^*^	1.367(0.569,2.165)	^**^	26.528(9.634,43.421)	^**^
Smoking habits (No)					23.964(3.139,44.788)	^*^
Alcohol consumption (No)	2.988(0.454,5.521)	^*^	0.698(0.020,1.376)	^*^		
4 doses of vaccination received (3 doses)	-3.088(-4.886,-1.290)	^**^	-0.631(-1.112,-0.150)	^*^		
R2	0.113		0.087		0.124	
Adj R2	0.099		0.075		0.109	
F	7.677	^***^	7.620	^***^	8.496	^***^
ISI						
constant	10.133(8.935,11.332)	^***^	1.07(0.758,1.383)	^***^	16.59(10.679,22.501)	^***^
HD(PD)	-1.754(-3.159,-0.35)	^*^				
Unable to work (Employed)	2.471(0.688,4.255)	^**^				
No family member living together (Less than 2)	3.237(0.443,6.032)	^*^				
Severe impact of the pandemic on household income (Slight)	3.664(1.371,5.958)	^**^	1.735(0.876,2.593)	^***^	37.775(21.539,54.012)	^***^
6–10 years of experience in dialysis (2–5 years)			-0.936(-1.57,-0.301)	^**^	-17.318(-29.314,-5.323)	^**^
Alcohol consumption (No)			1.138(0.406,1.87)	^**^	19.716(5.867,33.565)	^**^
4 doses of vaccination received (3 doses)	-1.843(-3.237,-0.449)	^*^				
R2	0.126		0.138		0.161	
Adj R2	0.115		0.128		0.143	
F	11.590	^***^	12.891	^***^	9.161	^***^

^*^ p<0.05; ^**^: p<0.01; ^***^: p<0.00..1.

For depression, higher scores were associated with lack of participation in religious activities, inability to work, severe pandemic-related household income loss, need for regular hospital visits for other major illnesses, and smoking. In contrast, independence in daily activities and completion of four COVID-19 vaccine doses were protective, corresponding to lower depression scores. These associations were consistent for both PWI and weighted scores.

For anxiety, significant risk factors included unemployment, availability of a separate room at home, severe income reduction, regular hospital visits for comorbidities, and alcohol consumption. Protective factors again included independent daily activity and full vaccination, both of which were consistently linked to reduced anxiety levels across PWI and weighted analyses.

For stress, significant risk factors were dialysis modality, unemployment, severe household income loss, and smoking or alcohol use. Independence in daily activities and four-dose vaccination were strongly protective.

For insomnia, risk factors included dialysis modality, inability to work, living alone, severe pandemic-related household income loss, dialysis duration of 6–10 years, and alcohol consumption. Conversely, patients who had received four vaccine doses reported milder insomnia symptoms.

Overall, socioeconomic disadvantage, comorbidity burden, and adverse health behaviors were consistently associated with greater psychological distress, whereas functional independence and complete vaccination served as robust protective factors.

## 4 Discussion

### 4.1 Overall prevalence and severity of psychological distress during the omicron wave

This study demonstrates that Taiwanese dialysis patients experienced substantial psychological distress during the Omicron outbreak, with insomnia the most prevalent symptom (54.7%), followed by stress (41.2%), anxiety (40.4%), and depression (14.3%). Compared with international reports, depression prevalence was notably lower than global averages (26–83%), particularly in regions severely affected by the pandemic such as South Asia and the Middle East, where rates often exceeded 70% (22–26). In contrast, anxiety prevalence was at the higher end of global estimates, while stress and insomnia rates were comparable to or slightly higher than those reported internationally (8, 10, 15, 23–30). These findings highlight that although Taiwan’s epidemic control was relatively successful, psychological distress remained a significant issue among dialysis patients.

The deterioration of mental health in this population reflects the interplay of biological frailty, multimorbidity, treatment dependency, and social isolation. Fear of contagion, disruptions in care, transportation barriers, and financial strain further compounded patients’ vulnerability. These results underscore the need for continuous psychological monitoring and targeted interventions for dialysis patients during public health crises (8, 10, 15, 22, 23, 28–34).

Interestingly, despite the unprecedented surge of Omicron cases in 2022, Taiwanese dialysis patients did not exhibit the dramatic escalation in distress observed in other countries. Mean Pandemic Worsening Index (PWI) scores remained modest, and depression prevalence was strikingly low compared with international figures. Several factors may explain this resilience, including strict infection-control protocols in dialysis centers, continuity of care through telehealth and psychosocial support, adaptive coping strategies developed by patients living with chronic illness, and relatively low national mortality rates. Collectively, these factors were associated with relatively lower the psychological impact of the pandemic and distinguish Taiwan as an outlier in the global context (8, 22, 27–29, 35–38).

### 4.2 Differences between HD and PD patients in psychological outcomes

This study found that PD patients experienced significantly greater psychological distress than HD patients during the Omicron outbreak, with higher levels of depression, anxiety, stress, and insomnia. These differences persisted even after adjusting for demographic and clinical characteristics, suggesting that the heightened burden among PD patients may be attributable to modality-specific factors rather than personal characteristics alone. The lack of daily medical monitoring, reduced peer interaction, and prolonged home confinement likely exacerbated social isolation and uncertainty, contributing to greater psychological vulnerability in PD patients (37).

By contrast, previous studies have shown that HD patients may also experience higher levels of distress, largely due to frequent hospital visits, increased exposure risk, transportation barriers, and older age with multiple comorbidities (10, 32, 34). Taken together, these findings highlight distinct stressors associated with each dialysis modality. For PD patients, interventions should focus on enhancing telemedicine access, psychosocial support, and virtual peer networks. For HD patients, ensuring safe transportation, maintaining continuity of care, and reinforcing infection-control measures are critical. Modality-specific psychosocial strategies are therefore essential to be associated with lower levels of psychological distress in dialysis populations during future public health crises.

### 4.3 Associations between personal characteristics and psychological symptoms

Stepwise regression identified several salient predictors of psychological distress among dialysis patients during the Omicron period. Risk factors included unemployment, substantial household income loss, smoking, alcohol consumption, and the need for regular follow-up due to other major illnesses. Each was independently associated with higher distress scores and showed consistent positive associations with both the Pandemic Worsening Index (PWI) and weighted composite scores, underscoring the critical influence of socioeconomic strain and adverse health behaviors on mental health outcomes. In contrast, two variables emerged as robust protective factors: independence in activities of daily living and completion of a four-dose COVID-19 vaccination series, both of which were consistently associated with lower distress levels and PWI scores. These findings suggest that functional independence and optimal vaccination coverage buffer the psychological impact of pandemic-related stressors in dialysis patients.

These results align with international evidence showing that socioeconomic disruption, comorbidity burden, and unhealthy behaviors exacerbate anxiety and depression among dialysis patients (8, 15, 16, 28, 39, 40). Conversely, vaccination and self-care ability have been repeatedly shown to protect against poor psychological outcomes, with fully vaccinated and functionally independent patients reporting greater resilience and more positive emotional states (29, 35).

Taken together, these findings emphasize the need for clinical teams to prioritize dialysis patients facing socioeconomic hardship, multiple comorbidities, and unhealthy lifestyle habits, and to implement early psychological screening and intervention. At the health system level, policies should actively promote vaccination and support patients in maintaining independence in daily living. For particularly vulnerable groups, multidisciplinary collaboration that integrates medical, psychological, and social resources will be critical to mitigating the mental health impact of future pandemics and other external stressors.

### 4.4 Limitation

This study has several limitations. First, its cross-sectional design restricts interpretation to a single time point, thereby precluding causal inference and limiting the ability to examine longitudinal changes in psychological health across different phases of the pandemic. Second, the sample was drawn exclusively from a single academic medical center in central Taiwan, which may limit representativeness and reduce generalizability to other regions or healthcare settings. Third, psychological distress was assessed using self-reported questionnaires, which are subject to social desirability and recall biases. Fourth, although adjustments were made for multiple potential confounding variables, several relevant factors were not assessed, including prior psychiatric history, history of COVID-19 infection, pre-existing sleep disorders, objective markers of kidney disease severity, coping strategies, family support, and cultural influences. The absence of these variables may have introduced residual confounding and could have influenced the observed associations with psychological outcomes. Finally, the Pandemic Worsening Index (PWI) used in this study was a self-developed measure. While it provided a useful proxy for subjective symptom exacerbation, its psychometric validity in dialysis populations remains to be established. Future studies should further evaluate its reliability and construct validity in larger and more diverse cohorts. Future studies should employ longitudinal designs and include larger, more diverse, and representative cohorts to strengthen external validity and improve causal inference.

Additionally, although the study aimed to recruit 200 HD and 200 PD patients, only 245 participants were ultimately enrolled (117 HD and 128 PD). The reduced sample size may have limited statistical power and representativeness, partly due to the constraints of infection control measures and patient safety concerns during the Omicron outbreak. Nevertheless, the achieved sample size provided sufficient statistical sensitivity for detecting group differences, as confirmed by *post hoc* power analysis.

## 5 Conclusion

Dialysis patients experienced a substantial psychological burden during the Omicron wave of COVID-19, with HD patients mainly affected by treatment-related risks and PD patients by reduced monitoring and social isolation. Socioeconomic disadvantage, unemployment, financial strain, comorbidities, and unhealthy behaviors were key predictors of distress, whereas full vaccination and independence in daily activities were identified as factors associated with lower levels of psychological distress. These findings highlight the need for routine psychological screening, timely supportive interventions, and programs that maintain functional independence. At the policy level, strengthening vaccination coverage and social safety nets will be essential. Multidisciplinary collaboration that integrates medical and psychosocial care is critical to mitigate psychological distress in dialysis populations during future public health crises.

## Data Availability

The original contributions presented in the study are included in the article/**Supplementary Material**. Further inquiries can be directed to the corresponding authors.
