# Supplementary figures and images for: Psychological distress among dialysis patients during the COVID-19 Omicron pandemic: risk and protective factors across hemodialysis and peritoneal dialysis
Source: Front Psychiatry. 2026 May 18;17:1710260. doi: 10.3389/fpsyt.2026.1710260 (PMC13223369; doi:10.3389/fpsyt.2026.1710260)

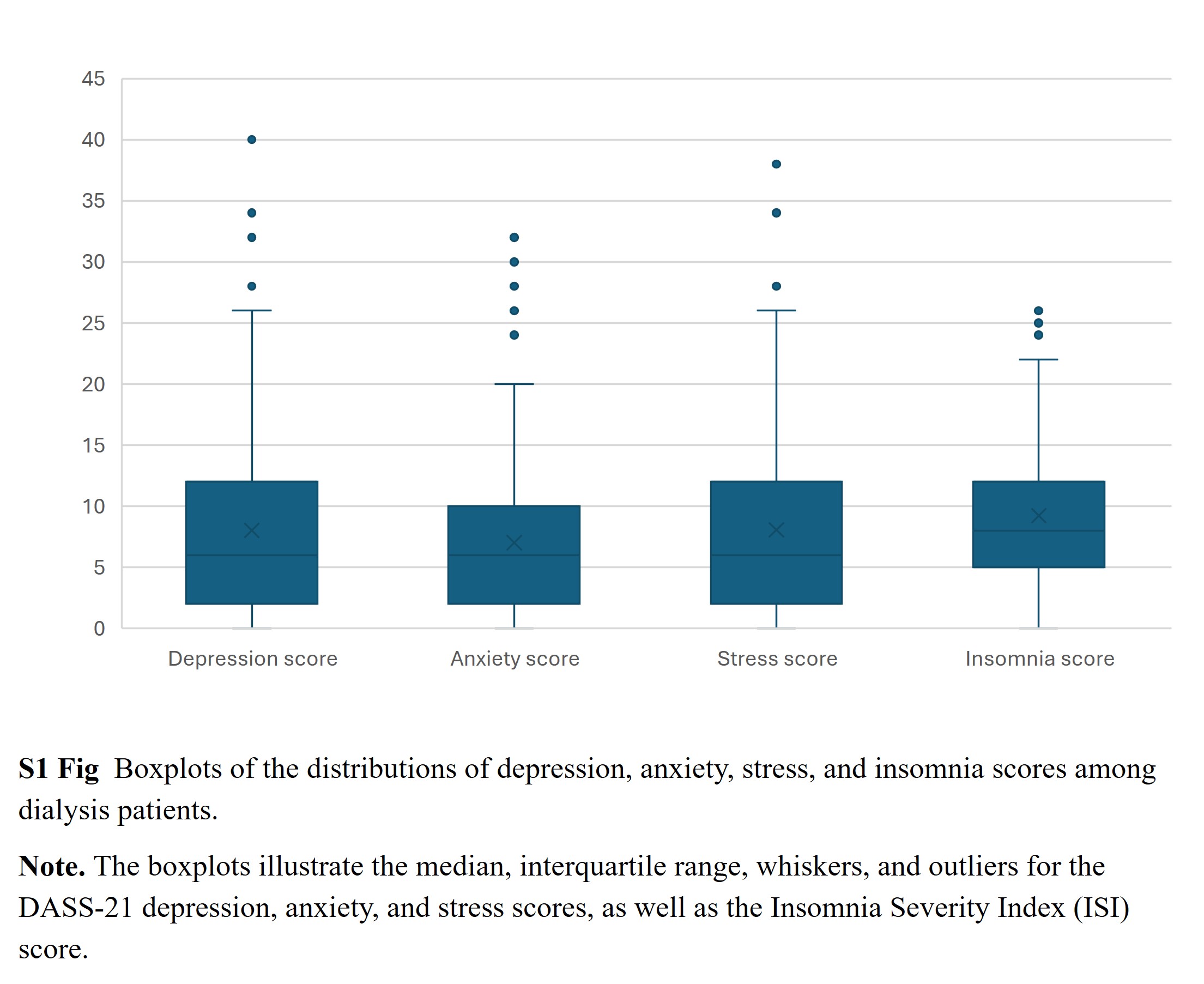

Supplement: Supplementary file 1 [file Image1.jpeg]

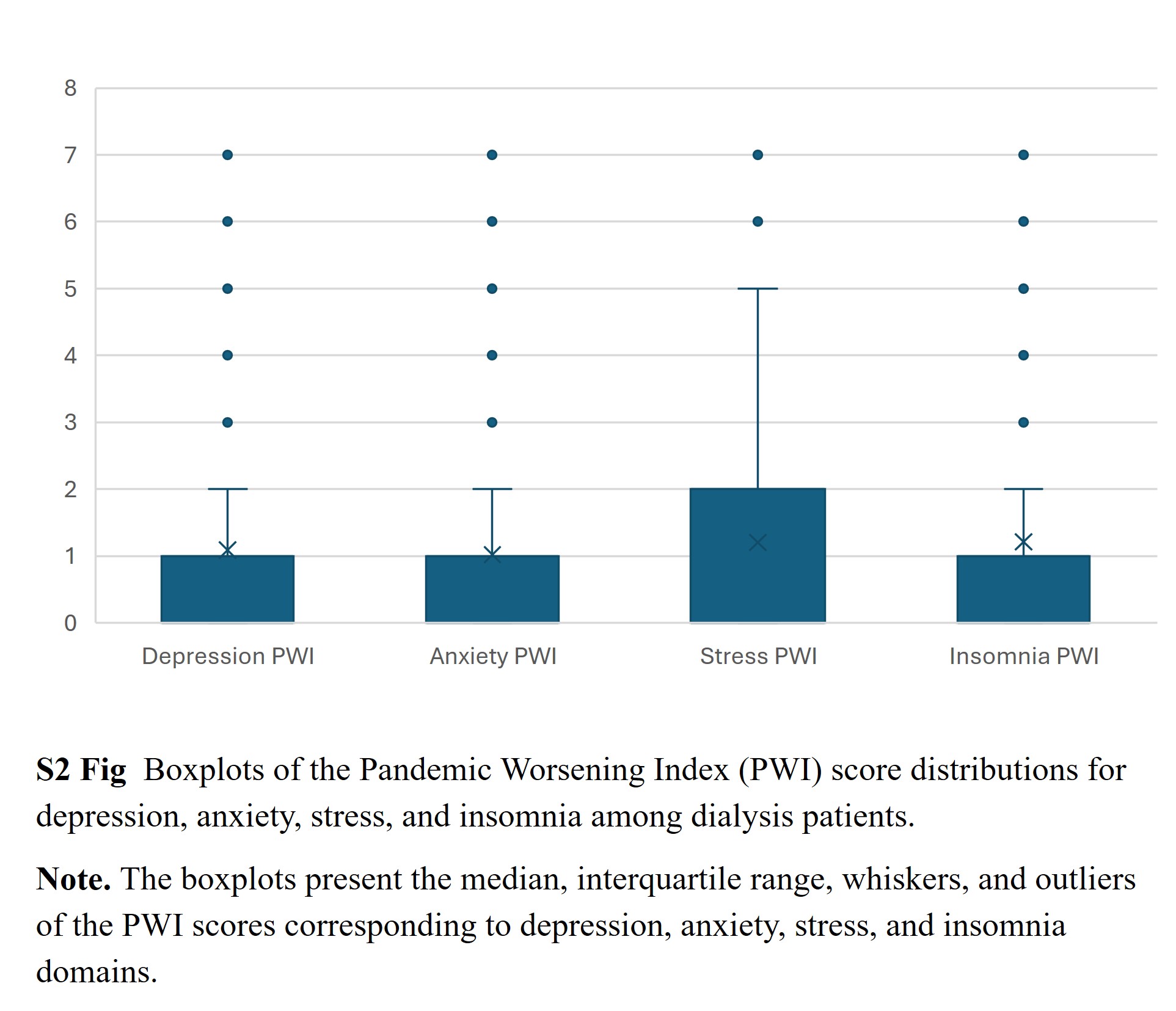

Supplement: Supplementary file 2 [file Image2.jpeg]
